# Unpacking overuse of androgen deprivation therapy for prostate cancer to inform de-implementation strategies

**DOI:** 10.1186/s43058-024-00576-x

**Published:** 2024-04-09

**Authors:** Ted A. Skolarus, Sarah T. Hawley, Jane Forman, Anne E. Sales, Jordan B. Sparks, Tabitha Metreger, Jennifer Burns, Megan V. Caram, Archana Radhakrishnan, Lesly A. Dossett, Danil V. Makarov, John T. Leppert, Jeremy B. Shelton, Kristian D. Stensland, Jennifer Dunsmore, Steven Maclennan, Sameer Saini, Brent K. Hollenbeck, Vahakn Shahinian, Daniela A. Wittmann, Varad Deolankar, S. Sriram

**Affiliations:** 1 VA HSR&D Center for Clinical Management Research, VA Ann Arbor Healthcare System, Ann Arbor, MI USA; 2 Department of Urology, Dow Division of Health Services Research, University of Michigan Medical School, Ann Arbor, MI USA; 3 Department of Internal Medicine, University of Michigan Medical School, Ann Arbor, MI USA; 4 Sinclair School of Nursing and Department of Family and Community Medicine, University of Missouri, Columbia, MO USA; 5 Department of Surgery, Rogel Cancer Center, University of Michigan, Ann Arbor, MI USA; 6 VA New York Harbor Healthcare System and NYU School of Medicine Departments of Urology and Population Health, New York, NY USA; 7 Surgical Service, VA Palo Alto Health Care System, Palo Alto, CA USA; 8 Department of Urology, Stanford University, Stanford, CA USA; 9 VA Salt Lake City Healthcare System, Salt Lake City, UT USA; 10 Department of Urology, University of California, Los Angeles, USA; 11 Academic Urology Unit, University of Aberdeen, Aberdeen, Scotland, UK; 12 Department of Urology, Massachusetts General Hospital, Boston, MI USA; 13 Ross School of Business, University of Michigan, Ann Arbor, MI USA; 14 Department of Surgery, Urology Section, University of Chicago, Chicago, USA

**Keywords:** Implementation, Behavior change, De-implementation, Low-value, Intervention, Behavioral theory, Complex health interventions, Theoretical domains framework, Discrete choice experiment, ADT Prostate cancer

## Abstract

**Background:**

Many men with prostate cancer will be exposed to androgen deprivation therapy (ADT). While evidence-based ADT use is common, ADT is also used in cases with no or limited evidence resulting in more harm than benefit, i.e., overuse. Since there are risks of ADT (e.g., diabetes, osteoporosis), it is important to understand the behaviors facilitating overuse to inform de-implementation strategies. For these reasons, we conducted a theory-informed survey study, including a discrete choice experiment (DCE), to better understand ADT overuse and provider preferences for mitigating overuse.

**Methods:**

Our survey used the Action, Actor, Context, Target, Time (AACTT) framework, the Theoretical Domains Framework (TDF), the Capability, Opportunity, Motivation–Behavior (COM-B) Model, and a DCE to elicit provider de-implementation strategy preferences. We surveyed the Society of Government Service Urologists listserv in December 2020. We stratified respondents based on the likelihood of stopping overuse as ADT monotherapy for localized prostate cancer (“yes”/“probably yes,” “probably no”/“no”), and characterized corresponding Likert scale responses to seven COM-B statements. We used multivariable regression to identify associations between stopping ADT overuse and COM-B responses.

**Results:**

Our survey was completed by 84 respondents (13% response rate), with 27% indicating “probably no”/“no” to stopping ADT overuse. We found differences across respondents who said they would and would *not* stop ADT overuse in demographics and COM-B statements. Our model identified 2 COM-B domains (Opportunity–Social, Motivation–Reflective) significantly associated with a lower likelihood of stopping ADT overuse. Our DCE demonstrated in-person communication, multidisciplinary review, and medical record documentation may be effective in reducing ADT overuse.

**Conclusions:**

Our study used a behavioral theory-informed survey, including a DCE, to identify behaviors and context underpinning ADT overuse. Specifying behaviors supporting and gathering provider preferences in addressing ADT overuse requires a stepwise, stakeholder-engaged approach to support evidence-based cancer care. From this work, we are pursuing targeted improvement strategies.

**Trial registration:**

ClinicalTrials.gov, NCT03579680

**Supplementary Information:**

The online version contains supplementary material available at 10.1186/s43058-024-00576-x.

Contributions to the literature

- Using a behavior specification framework to characterize androgen deprivation therapy overuse is novel and important given historical and current opportunities to improve evidence-based castration practices for men with prostate cancer.
- Examining stated preferences for stopping overuse in specific clinical scenarios and then clarifying theory-informed beliefs across the Capability, Opportunity, and Motivation domains of the Behavior Change Wheel Model adds to intervention design and tailoring literature, supporting deeper investigation into causal mechanisms of implementation interventions.
- Reporting the use of theory-informed discrete choice experiments in implementation science adds to expanding methodological resources for investigators.

## Introduction

Up to one-third of men diagnosed with prostate cancer will be exposed to androgen deprivation therapy (ADT), a form of chemical castration administered by long-acting injection, at some point during cancer survivorship [1]. The use of evidence-based ADT (e.g., in combination with radiotherapy for high-risk localized prostate cancer and metastatic disease) is associated with improved survival and symptomatic relief [2]. On the other hand, using ADT in clinical scenarios where the evidence and value base are limited (e.g., monotherapy in localized prostate cancer and non-metastatic biochemically-recurrent disease) [3–5] also occurs resulting in more harm than benefit, termed ADT overuse. For instance, among men not receiving definitive treatment for localized prostate cancer, 20% and 40% of those with intermediate- and high-risk disease may be treated with non-curative ADT monotherapy [6]. Addressing overuse is important because of the potential harms of castration with ADT including diabetes, cardiovascular disease, and osteoporosis, among others [7]. More broadly, addressing overuse is key to international Choosing Wisely efforts for improving the value of cancer care [8].

The actual behaviors and scenarios underpinning ADT overuse remain unclear leading to non-existent or poorly informed improvement strategies. It is necessary to understand these scenarios before targeted improvement strategies to limit ADT overuse can be pursued. For example, which clinicians order ADT, when is it ordered, in what clinical scenarios, and how confident are providers in stopping ADT overuse? Indeed, specifying behaviors supporting ADT overuse, better understanding barriers to stopping ADT overuse, and gathering provider preferences when it comes to addressing ADT overuse requires a stepwise, stakeholder-engaged approach to support evidence-based prostate cancer care.

For these reasons, we conducted a behavioral theory-informed survey study to understand ADT overuse in localized prostate cancer with the goal of informing improvement strategies focused on de-implementation. We clarified behaviors related to ADT use within a national healthcare system, including in the overuse scenario of ADT monotherapy for clinically localized prostate cancer. Using behavior specification and behavior change frameworks, coupled with a discrete choice experiment (DCE) provider survey to gather preferences for tradeoffs across implementation strategies, we laid the groundwork for theory-informed strategies targeting ADT overuse, as well as provide guidance to others invested in addressing overuse of cancer care using a stepwise, stakeholder-engaged approach.

## Methods

The overarching aim of this multipronged research study was to contextualize ADT overuse and inform strategies to de-implement it using a behavioral theory-informed survey and DCE. To accomplish this aim, we used preliminary data from our theory-based qualitative findings [9] to develop and field a national survey of urology providers.

Our overall theory-informed survey had 3 main sections: (1) ADT overuse behavior specification, (2) barriers to and facilitators of ADT overuse, and (3) a DCE to quantitatively assess provider preferences for tailoring de-implementation strategies targeting ADT overuse. In the first section, we wished to gain an understanding of the target behavior, i.e., ADT overuse in the scenario of monotherapy for localized prostate cancer. To clearly identify behaviors involved in ADT overuse and inform behavior change strategies, we used the Action, Actor, Context (Scenario), Target, Time (AACTT) framework for behavior specification [10]. This rigorous behavioral specification framework helped clarify who needs to do what differently and under what circumstances to decrease ADT overuse. The Target in this case (i.e., to or for whom the action was being performed) was a theoretical patient receiving ADT monotherapy for localized prostate cancer, i.e., ADT overuse. In this clinical scenario, we asked respondents to assess (1) how often they started ADT monotherapy in localized prostate cancer patients, (2) how often patients already on ADT for localized prostate cancer presented to their practice, and (3) whether they had ever stopped or recommended stopping ADT monotherapy for localized prostate cancer (yes, no). For those responding *yes* and *probably yes *to stopping ADT in response to the following question: “For patients who come to your practice and are already on ADT monotherapy for localized prostate cancer, would you recommend stopping ADT?” we queried their initial follow-up intervals and clinical assessments (1, 3, 6, and 12 months) across the following clinical resources: office visit, symptom assessment, physical examination/digital rectal examination, PSA level) further adding to Context/Scenario and opportunities for substitution [11].

Our second survey section evolved from our prior qualitative investigation [9] grounded in the Theoretical Domains Framework (TDF) [12] and the Behavior Change Wheel’s Capability, Opportunity, Motivation–Behavior (COM-B) Model [13] to better understand provider perspectives on ADT for localized prostate cancer, and barriers to and facilitators of addressing ADT overuse through de-implementation. We conducted 20 semi-structured interviews with urology providers and discovered three provider types: (1) those who never prescribed ADT monotherapy for localized prostate cancer (i.e., no overuse), (2) providers who were willing under some circumstances to prescribe ADT for localized disease (e.g., patient preference), and (3) providers who deemed ADT monotherapy a reasonable treatment option for localized disease (e.g., based on experience). Further, by mapping the qualitative findings to Capability, Opportunity, and Motivation domains we conceptualized possible intervention opportunities to address ADT overuse. For example, we found interpersonal skills (i.e., Capability–Psychological) and social influences (Opportunity–Social) were important facilitators of stopping ADT leading to our survey statement selections described below. In this manner, we were particularly interested in respondent agreement with COM-B domain statements according to whether they would or would *not* stop ADT monotherapy for patients with localized prostate cancer presenting to their practice.

We used a 5-point Likert scale (from strongly disagree to strongly agree) to measure agreement with 7 domain statements addressing TDF and COM-B domains. Examples include “I find that patients are worried about the effect that stopping ADT will have on their cancer” (TDF domain: beliefs about consequences; COM-B Motivation–Reflective, Opportunity–Social (patient influence)) and “I do not have adequate time for discussion about ADT” (TDF domain: environmental resources; COM-B Opportunity–Physical). We hypothesized respondents who would *not* stop ADT in their practice would be more likely to agree with “I find talking about stopping ADT challenging” (TDF domain: skills—interpersonal; COM-B Capability–Psychological). Based on the COM-B, this would map to psychological capability and guide our intervention towards behavior change techniques to improve capability such as modeling the behavior or scripts to help with ADT overuse conversations. This was a key step towards unpacking our specified behavior of interest—ADT overuse in localized prostate cancer—and important corresponding behavioral domains and potential levers, particularly among those providers who would *not* stop ADT overuse. These findings, coupled with our DCE, would provide the foundation for subsequent de-implementation strategy selection and tailoring to address ADT overuse.

**Fig. 1:**
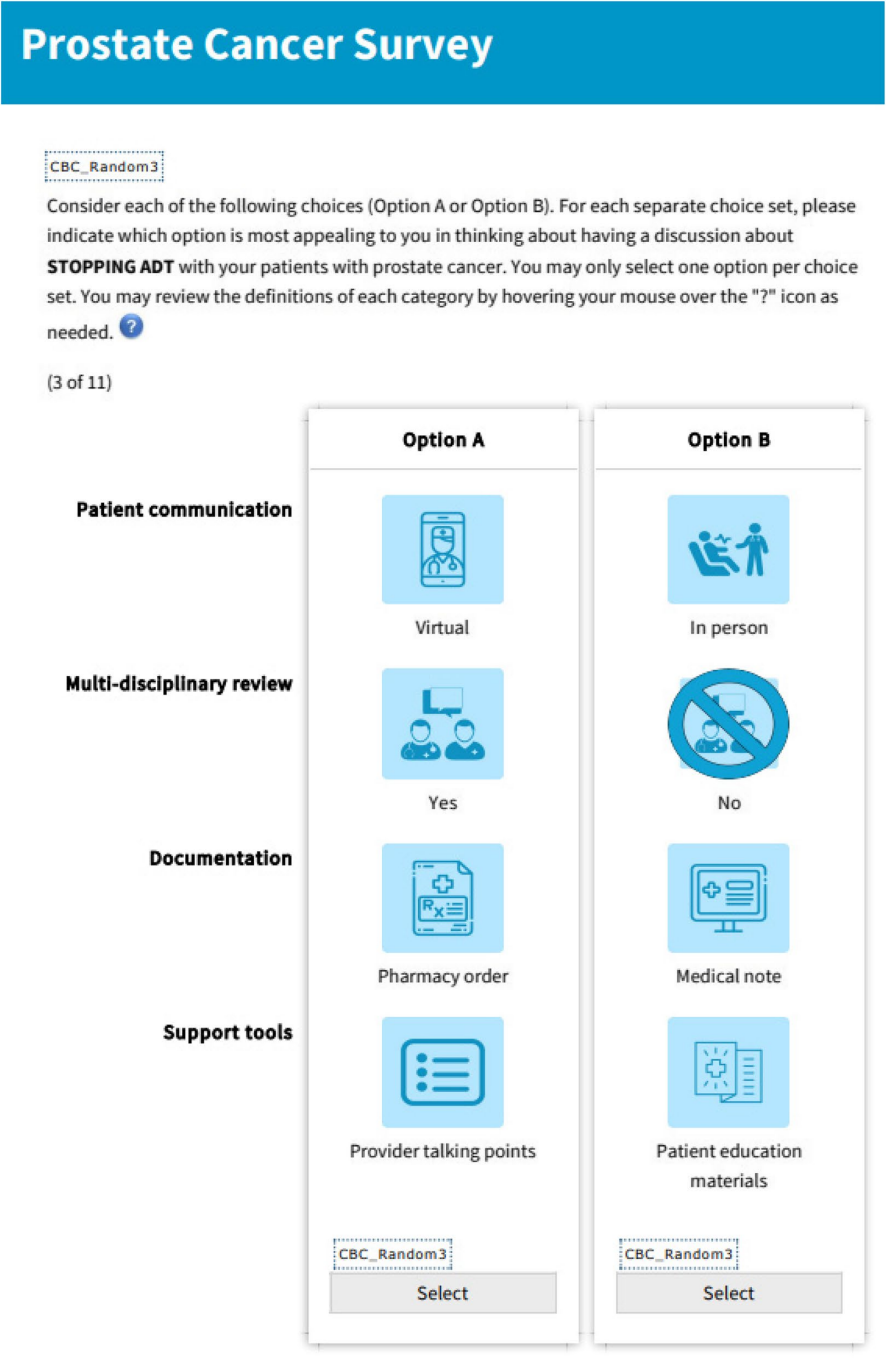
Example choice set from the DCE survey

The third section of the overall survey included a DCE grounded in COM-B domains to quantitatively assess provider preferences for tailoring de-implementation strategies targeting ADT overuse. Discrete choice experiments are survey-based tools to facilitate priority setting and drive strategy development in industry, with increasing applications to health care and implementation [14–16], such as using a DCE to understand and weight colorectal cancer screening preferences to guide patient screening strategies [17]. Using a similar approach, we conducted a DCE to elicit provider preferences with respect to strategy selection and tailoring to support de-implementation of ADT overuse. We asked respondents to react to hypothetical choice sets based on a combination of attributes (characteristics of strategies addressing ADT overuse) and levels (descriptors of each attribute). In order to avoid respondent overload when evaluating the choice sets, and based on the literature [18], we elected to use four attributes with two levels each, yielding a total of 16 profiles. The four de-implementation strategy attributes and the corresponding levels were informed by our prior qualitative findings and behavioral specification needs (Table 1). Ascertaining the relative strengths of provider preferences with respect to these strategy attributes and levels through a quantitative DCE would enable us to select preferred behavior change techniques and direct de-implementation tailoring efforts. For example, we hypothesized that respondents with lower confidence discussing ADT may prioritize Multidisciplinary Review. If this is true, a strategy grounded in social opportunity such as peer support or review is likely to be effective in reducing ADT overuse. A sample discrete choice task is illustrated in Fig. 1.

**Table 1:** Attribute characteristics for the discrete choice experiment

**Attribute**	**Description**	**COM-B domain**	**Levels**
Patient communication	Speaking with patients about the risks and benefits of ADT	Capability–Psychological	In-person Virtual
Multidisciplinary review	Consulting with colleagues (e.g., urologic oncologist, medical oncology) prior to the patient appointment, or not, to assist with de-implementation decisions regarding ADT overuse	Opportunity–Social (provider)	Yes No
Documentation	Documenting ongoing ADT treatment decisions in a pharmacy order or a medical note	Opportunity–Physical	Pharmacy order Medical note
Support tools	Support tools to aid with conversations to include provider talking points or patient education materials regarding ADT overuse	Opportunity–Social (patient support)	Provider talking points Patient education materials

The final survey section contained demographic information regarding the provider and their practice. We piloted the survey among our study team for final recommendations and fielded the survey to the Society of Government Service Urologists email listserv comprised of Veterans Affairs and active military urologists and providers across the USA (*n* = 625), with and without academic affiliations. All participants were asked to complete the entire survey, including all DCE items, regardless of responses regarding stopping ADT. We sent the initial email invitation and two reminder invitations in December 2020, including a $50 incentive for completing the survey.

### Statistical analyses

First, we used descriptive statistics to characterize behavioral specification responses from our first survey section. We grouped respondents by our primary stratification variable, i.e., “yes/probably yes” and “no/probably no” for stopping ADT in the scenario of a localized prostate cancer patient. We used univariate statistics to compare the groups according to respondent demographics. Next, we used univariate statistics to compare the groups according to agreement with each of the seven statements addressing TDF and COM-B domains. Finally, to better understand domains underpinning provider decisions to stop prescribing ADT, we estimated a multivariable logistic regression where “yes/probably yes” was the dependent variable and dichotomized Likert scale responses of “strongly disagree/disagree/neutral” and “agree/strongly agree” across the statements were the independent variables. We grouped “neutral” with “strongly disagree/disagree” as the statements reflect an active component (e.g., desire to use other treatments than ADT) that align more closely with the disagree than agree motivational factors. We considered this dichotomous separation to better align with the likelihood of influence on stopping ADT.

For the DCE analysis, we estimated the partworths, or attribute importance scores, using a standard multinomial logit model and a hierarchical Bayes version of the multinomial logit model. While the former assumes that the coefficients (and partworths) are homogenous across providers, the latter helped us explore heterogeneity across providers. Based on these partworths, we computed the relative importance of each attribute *j* as ${r}_{j}=\frac{{{\text{Importance}}}_{j}}{\sum_{j{\prime}}^{4}{{\text{Importance}}}_{j{\prime}}}$. We deduced the relative importance of each attribute by considering the maximum difference in partworths between various levels within an attribute. This enabled us to examine the relative importance of each of the four attributes (communication, documentation, multidisciplinary review, support materials). Furthermore, using the individual level partworths from the hierarchical Bayes version of the multinomial logit model, we were able to explore differences in the relative importance of attributes based on respondent characteristics. We performed our DCE analysis using the built-in analysis package in Sawtooth Software’s Lighthouse Studio [19].

This study was approved by the VA Ann Arbor Healthcare System and the University of Michigan Institutional Review Boards. Analyses were conducted using Sawtooth and R with the probability of a type I error set at 0.05, and all testing was two-sided. We used the STROBE reporting checklist for cross-sectional studies included in Additional file 1.

## Results

### Behavioral specification for ADT use and overuse

Our survey was completed by 84 out of 625 respondents (13% response rate). We found most respondents indicated “yes” (19%) and “probably yes” (54%) to our primary question to define ADT overuse: “For patients who come to your practice and are already on ADT monotherapy for localized prostate cancer, would you recommend stopping ADT?” However, 27% of providers indicated they would be unlikely to recommend stopping ADT indicating “probably no” and “no.” When asked about follow-up after stopping ADT, respondents commonly indicated 3 months for interval symptom assessment (83%), office visit (81%), digital rectal examination (63%), and PSA (79%). Provider demographics stratified by responses are shown in Table 2.

**Table 2:** Respondent demographics according to stopping ADT overuse

	For patients who come to your practice and are already on ADT monotherapy for localized prostate cancer, would you recommend stopping ADT?		
	Yes/probably yes (*n* = 61)	No/probably no (*n* = 23)	*p*-value
Gender (*n*, % male)	49, 80%	22, 96%	0.01
Race (*n*, %)			0.63
White	47, 77%	18, 78%	
Non-White	9, 15%	2, 9%	
Prefer not to disclose	5, 8%	3, 13%	
Years in practice (*n*, %)			0.16
Less than 5 years	13, 21%	5, 22%	
5–10 years	13, 21%	3, 13%	
11–15 years	12, 20%	0	
More than 15 years	23, 38%	15, 65%	
VA practice (*n*, %)			0.04
Full time	13, 21%	3, 13%	
Part time	23, 38%	4, 17%	
None	25, 41%	16, 70%	
Academic affiliation (*n*, % yes)	40, 66%	13, 57%	0.61
Fellowship training in urologic oncology (*n*, % yes)	11, 18%	4, 17%	1
Do you treat patients with metastatic prostate cancer using ADT (*n*, % yes)	51, 84%	19, 83%	1
Does your practice conduct prostate cancer clinical trials (*n*, % yes)	29, 48%	9, 39%	0.66
How confident are you discussing the risks and benefits of ADT monotherapy for patients with localized prostate cancer? (*n*, %)			0.27
Not at all confident	1, 2%	0	
A little/somewhat confident	11, 18%	7, 30%	
Quite/extremely confident	49, 80%	16, 70%	
Have you ever stopped prescribing ADT as monotherapy for one of your patients with localized prostate cancer? (*n*, %)			
Yes	47, 77%	16, 70%	0.67
I prefer to emphasize the following when communicating with patients about stopping ADT for localized prostate cancer: (*n*, %; [57 of 61 first column responses])			0.62
Harms of continuing ADT	21, 37%	10, 45%	
Neutral	11, 19%	5, 23%	
Benefits of stopping ADT	25, 44%	7, 32%	

Respondents clarified ADT use according to the AACTT as follows. We found the Action of ordering ADT was primarily conducted by the urologist (i.e., Actor) either most of the time (45%) or always (20%). Nearly half of respondents (49%) indicated urology nurse practitioners or physician assistants ordered ADT in their practice at least sometimes, with slightly less indicating urology residents or fellows were involved in ordering ADT (44%). Medical oncology and radiation oncology “sometimes” ordered ADT among 63% and 56% of respondents, respectively. With respect to the Context/Scenario of ADT dosing, respondents were split between 3 (55%) and 6 (42%) month doses. PSA testing was similarly split at 3 (49%) and 6 (48%) month frequencies. Clinic visits for patients on ADT for prostate cancer were less frequent, with 6-month (54%) intervals cited as the most common. When asked how patients were notified of their PSA results, most respondents indicated this was done during a clinic visit (52%), followed by telephone (21%), and patient portal (14%). According to respondents, we identified variable Time of ADT ordering in the electronic medical record ranging from the day of injection for nearly half of respondents (47%), to greater than a week before the injection (17%). One in five respondents indicated they did not know of a specific pattern to ADT ordering.

### Behavioral theory-guided assessment of barriers to and facilitators of stopping ADT overuse

As shown in Table 3, our multivariable model identified two COM-B statements significantly associated with a lower likelihood of stopping ADT overuse. Respondents in agreement with the following statement: “I find patients are worried about the effect stopping ADT will have on their cancer” were less likely to stop ADT overuse indicating COM-B Motivation–Reflective (provider), Opportunity–Social (patient) were relevant to strategy selection and tailoring (adjusted odds ratio (aOR) 0.22, 95% confidence interval (CI) 0.05–0.79). In addition, respondents in agreement with the statement: “I want to give ADT recommendations consistent with those of my peers” were also less likely to stop ADT overuse indicating COM-B Opportunity–Social (provider) may also play an important role in intervention tailoring (aOR 0.12, 95% CI 0.01–0.67). The remainder of aORs were positive though non-significantly associated with a respondent preference of stopping ADT overuse.

**Table 3:** Multivariable model of COM-B domain statements associated with the likelihood of stopping ADT overuse

**Survey statements**	**TDF domain**	**COM-B domain(s)**	**Adjusted odds ratio (95% CI)**	***p*-value**
I find patients are worried about the effect stopping ADT will have on their cancer	Beliefs about consequences	Motivation–Reflective (provider) Opportunity–Social (patient)	0.22 (0.05–0.79)	0.03
I want to give ADT recommendations consistent with those of my peers	Social influence	Opportunity–Social (provider)	0.12 (0.01–0.67)	0.04
I have concerns about side effects and castration resistance in patients with long-term use of ADT	Beliefs about consequences	Motivation–Reflective	2.42 (0.60–9.95)	0.21
I put a lot of weight on guideline recommendations regarding use of ADT as monotherapy (e.g., AUA or NCCN)	Environmental resources	Opportunity–Physical	2.73 (0.50–15.90)	0.24
I do not have adequate time for discussion about ADT	Environmental resources	Opportunity–Physical	2.82 (0.31–65.40)	0.41
I find talking about stopping ADT challenging	Skills–interpersonal	Capability–Psychological	2.03 (0.35–14.30)	0.44
I like to consider options other than ADT to manage patients with localized prostate cancer (e.g., definitive treatment, watchful waiting)	Knowledge	Capability–Psychological	1.00 (0.01–100)	0.99

### Discrete choice experiment

We report the results from the multinomial logit model and hierarchical Bayes version in Table 4. Our results suggest that in-person (vs. virtual) communication with patients, presence of multidisciplinary review (vs. not having one), documentation in the form of a medical note (vs. pharmacy order), and support tools in the form of patient education materials (vs. provider talking points) would be more effective in stopping ADT overuse as monotherapy for localized prostate cancer. These results were consistent across both estimation approaches.

**Table 4:** Estimates of partworths^a^ from the DCE

**Attribute level**	**Partworths–Logit**	**Parthworths–Hierarchical Bayes**
**Patient communication**		
In person	0.529 (0.049)	1.123 (0.073)
Virtual	− 0.529 (0.049)	− 1.123 (0.073)
**Multidisciplinary review**		
No	− 0.299 (0.046)	− 0.591 (0.115)
Yes	0.299 (0.046)	0.591 (0.115)
**Documentation**		
Pharmacy order	− 0.360 (0.048)	− 0.766 (0.056)
Medical note	0.360 (0.048)	0.766 (0.056)
**Support tools**		
Provider talking points	− 0.140 (0.045)	− 0.213 (0.047)
Patient education materials	0.140 (0.045)	0.213 (0.047)
**Relative importance of attributes (hierarchical Bayes only)**		**Average importance (%) (standard deviation)**
Patient communication		35% (18.31)
Multi-disciplinary review		28% (17.85)
Documentation		24% (13.56)
Support tools		13% (8.45)

^a^Estimates of raw utilities (partworths) that were zero-centered within each attribute (i.e., the sum of the partworths for different levels within each attribute sum to 0)

**Fig. 2:**
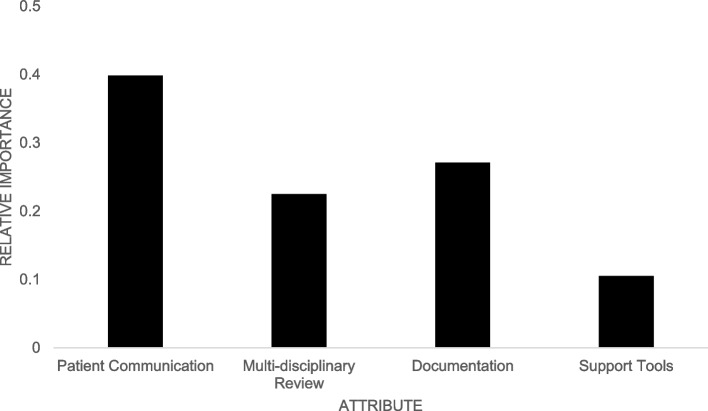
Discrete choice experiment relative importance of communication, multidisciplinary review, documentation, and support tools from logit estimation

The relative importance of attributes among all respondents based on logit analysis is illustrated in Fig. 2. These results suggest patient communication was the most important attribute, followed by documentation and multi-disciplinary review. The availability of support tools was the least important attribute. For hierarchical Bayes, we report the average importance values across all providers in Table 4. These results were consistent with those from the logit analysis. The only difference was the switch in the relative positions of multidisciplinary review and documentation. In this regard, it is worth noting that both the logit and hierarchical Bayes analysis suggested these two attributes were similar in terms of their relative importance in stopping ADT overuse for localized prostate cancer.

**Fig. 3:**
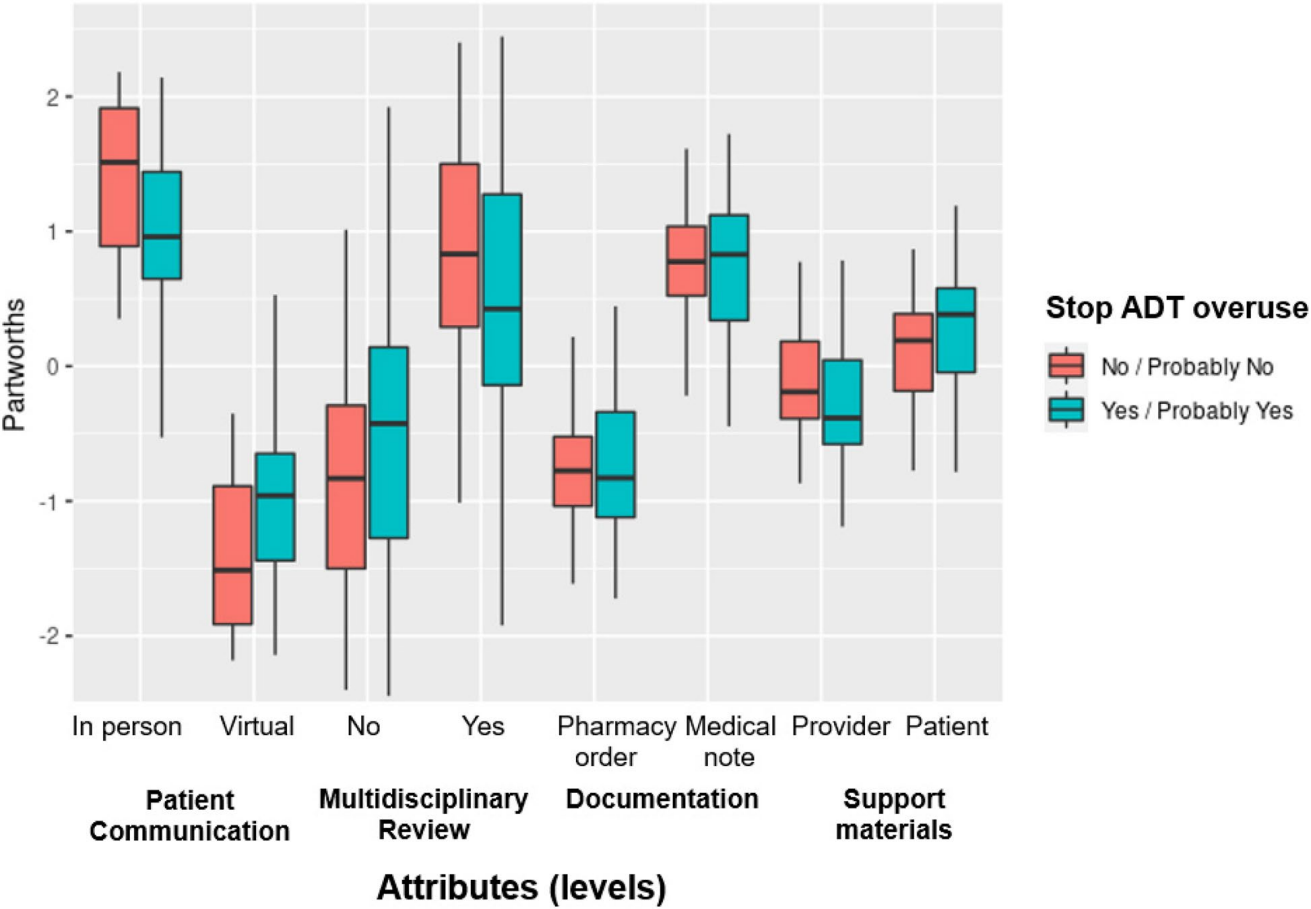
Discrete choice experiment partworths utilities for communication, multidisciplinary review, documentation, and support tools according to whether provider respondents would or would *not* stop low-value ADT

As noted above, the hierarchical Bayes analysis helped us explore heterogeneity in the partworths and relative importance across different subgroups of providers. In particular, we considered subgroups defined by whether respondents would or would *not* stop ADT overuse. We present these results in Fig. 3. When we examined the attribute levels underpinning provider preferences across our four attributes using partworths, we found a strong preference for in-person patient communication compared to virtual options. This is true for all providers, irrespective of whether they would and would *not* stop low-value ADT (Fig. 3). We also found a preference for multidisciplinary review prior to the appointment among all respondents, with attenuated preference differences across respondent groups. There were similar preferences across groups with respect to documenting ADT overuse treatment decisions in the medical record rather than in pharmacy orders. Lastly, we identified relatively neutral preferences across groups regarding provider talking points vs. patient education materials. Taken together, our DCE demonstrated provider respondents tended to prefer in-person communication, with multidisciplinary review and medical record documentation, with little preference regarding provider scripting versus patient support materials.

## Discussion

Using a survey grounded in behavior specification and theory-based behavior change, coupled with a DCE to gather provider preferences, we better define the actual behaviors and scenarios underpinning ADT overuse and clarified characteristics and stated preferences for addressing it. While most respondents would stop ADT overuse among localized prostate cancer patients presenting to their practice, 1 in 4 would not. This coupled with population-based data indicating older men with higher-risk prostate cancer are more likely treated with ADT monotherapy [6], confirms interventions are warranted to minimize ADT overtreatment and its harms. Moreover, we found only half of ADT orders were placed on the day of a visit indicating electronic health record opportunities to discontinue or renew ADT prior to the clinical encounter. Indeed, our rigorous behavior specification using the AACTT framework helped clarify who (Actor) needs to do what (Action), when (Timing), and in what situations (Context/Scenario) when it comes to ADT overuse. Defining the problem of ADT overuse in these behavioral terms enables the use of the theory-informed Behavior Change Wheel approach [13] to guide behavior change technique selection and de-implementation strategy development.

We found several unique aspects of ADT-related behaviors, including ADT overuse, clarified by our study. First, we identified ADT was administered at different time intervals (e.g., 1, 3, and 6 months) and by different ordering provider specialties (urology, radiation, and medical oncology) adding complexity to de-implementation strategies. On the one hand, this might allow for multiple opportunities to address ongoing ADT overuse. On the other hand, such intermittent and variable use of a long-acting chemical castration agent across provider types creates challenges for operationalizing standardized in-clinic interventions necessitating higher degrees of scenario assessment and strategy tailoring. This scenario is unique to chemical castration with serial ADT injections versus a single surgical castration event that has largely fallen out of favor (i.e., orchiectomy) [20]. Second, we discovered a disconnect between when ADT was ordered and when clinic visits occurred with only half of providers indicating ADT was ordered on the day of the injection. Ordering ADT before clinic visits may create clinical inertia to continue prescribing despite potentially favorable PSA levels or patient-reported worsening of ADT side effects that would support de-implementation. This could also imply patients may present for ADT injections without a clinical evaluation leading to missed opportunities and additional months of castration. Indeed, once patients are on board the “ADT train” it appears they may not be able to get off until a subsequent clinic visit. This disconnect between the ADT ordering process and clinical assessment, i.e., asynchronous ADT ordering, should be considered when designing de-implementation interventions. Third, among those provider respondents who had experienced stopping ADT overuse, follow-up preferences included an interval symptom assessment, PSA, and office visit at 3 months. This finding supports coupling a 3-month follow-up appointment as a substitution for ADT overuse to align de-implementation with ongoing clinical care [11, 21, 22].

Using the COM-B Model [23] in our survey, we identified the Social Opportunity and Reflective Motivation domains as relevant to de-implementation. Our Social Opportunity findings were two-fold. First, respondents who would *not* stop ADT overuse were more concerned about patient worry regarding the impact of stopping ADT on their cancer. This could support opportunities for patient interventions to dispel concerns (e.g., patient education about the lack of benefit of ADT in this scenario, activation), including the ability to restart ADT if there were signs of cancer progression. Second, respondents less likely to stop ADT overuse also wanted to practice like their peers. This counterintuitive finding may relate to how our question was worded, i.e., “for patients referred to your practice…” If these respondents desired to practice like most of their peers, rather than referring providers overusing ADT, they would stop ADT as confirmed in our survey and prior qualitative findings. There is also a broader component of “peers” perhaps reflected in guidelines, e.g., meta-peers. Taken together, our Social Opportunity findings support ADT overuse de-implementation opportunities at both the patient and provider levels.

Another important consideration from our study indicated Reflective Motivation may also be at play when it comes to stopping ADT overuse, primarily through the beliefs about consequences construct, with beliefs about consequences of stopping ADT and disease progression perceived by patients favoring continued ADT use, and on the other hand, concerns about ADT side effects and castration resistance with long-term use favoring de-implementation. Although the latter was not statistically significant potentially due to sample size constraints, respondents more likely to stop ADT generally agreed with these concerns. This interplay supports targeting Reflective Motivation in messaging interventions to both patients and providers when seeking to intervene on ADT overuse.

Finally, our DCE highlighted the feasibility of using this important approach and differentiating responses across providers who would or would *not *stop ADT overuse. This potentially generalizable approach quantified the relative importance and utilities of preferences for de-implementation strategy design. First, in a scenario where de-implementation was presented, we identified a strong preference for in-person communication compared with virtual options. Over the past three years, however, virtual options have launched into mainstream practice due to COVID and enabling technologies leaving this option subject to change over time [24–26]. We found multidisciplinary review was preferred regardless of ADT practices although potentially more so among those less likely to stop ADT overuse. This might be akin to presenting complex cancer cases at tumor board for multidisciplinary support, in this case for stopping cancer care overuse, and aligns with our Social Opportunity findings. This also aligns with our prior qualitative findings of provider confidence when it comes to stopping ADT overuse [9]. We identified a preference towards documenting ADT decision-making in medical notes rather than in the pharmacy order. This is not surprising given medicolegal considerations and fear of litigation particularly when stopping a cancer treatment even when it is overused. Lastly, there was little relative preference for provider talking points vs. patient support materials to aid in ADT decision-making.

This deep dive into provider behavior and preferences when addressing ADT overuse provides supporting empirical data to guide de-implementation strategy selection and tailoring. Nonetheless, there are some limitations to consider. First, our response rate for this provider survey was low despite multiple invitation reminders and financial incentives. While the internal validity of the findings (i.e., stratification by whether a provider would or would *not* stop ADT) may be relatively strong and a potential model for designing interventions according to stated overuse decisions, a greater limitation is to external validity to the broader urologist community given the inclusion of government service urologists. Second, our sample size limited the statistical significance of our findings, particularly for demographics across provider preferences. We did identify some differences with trends across demographics, COM-B domains, and attributes, however, and our DCE used rigorous methods to guide our preference determinations for the next steps. Moreover, there could also be self-selection bias in that providers responding to the survey were more likely to be guideline concordant and not overuse ADT. Given 1 in 4 providers would not stop ADT overuse, we were able to stratify and examine contrasting responses and potential intervention implications. Larger scale surveys using this methodology may be used to validate and expand our findings. Third, the widespread variability in ADT practices across providers and clinics could limit the specificity of these findings to inform de-implementation strategies. Nonetheless, recognizing the complexities underpinning behavior specification and ADT decision-making contributes to generalizable knowledge. Lastly, while we developed statements aligned with each COM-B domain, we also acknowledged potential overlap with different domains. For instance, with respect to our guidelines statement as an environmental resource (Opportunity–Physical), this could be perceived as beliefs about the consequences of following the guidelines for patient outcomes (Motivation–Reflective), and even knowledge of the guidelines (Capability–Psychological). We chose this as an environmental resource, i.e., readily available care recommendations, reflecting broader environmental resources to guide care. For the statement regarding a challenging conversation, we classified this as Capability–Psychological based on the need for interpersonal skills informed by our prior work though recognize this might be related to professional confidence, beliefs about capability, and hence, Motivation–Reflective also noted as a facilitator to stopping ADT. Ultimately, the design and tailoring of pilot interventions need to take these considerations into account.

Using the guidance provided by this study, we are tailoring two competing de-implementation interventions to address ADT overuse, each operating at different levels of healthcare delivery and designed to be tested against each other [27]. The design and tailoring of our interventions will be focused on the AACTT specifications, the COM-B behavioral determinants, and the prioritization of the attributes and levels clarified, at least in part, by this study. The first is a clinical reminder order check embedded in the electronic medical record, acting as a blunt, organizational-level intervention when ordering ADT in overuse scenarios (e.g., localized prostate cancer). The second is a medical record note with supporting provider scripts, and patient support materials targeting the dyadic relationship among providers and patients receiving ADT overuse. We will use the Implementation Research Logic Model [28] to link the determinants, implementation strategies, hypotheses, possible causal mechanisms, and outcomes in preparation for conducting a randomized comparative effectiveness de-implementation trial addressing ADT overuse.

## Conclusions

Our study aimed to identify behaviors and scenarios underpinning ADT overuse. Using a behavioral theory-informed survey, we discovered providers less likely to stop ADT overuse had greater concern about patient worry and were more interested in providing ADT consistent with peers, informing de-implementation strategy selection and tailoring. We found respondents tended to prefer in-person communication, with multidisciplinary review and medical record documentation, and little preference regarding provider scripting and patient support materials when it comes to addressing ADT overuse. Specifying behaviors supporting ADT overuse and gathering provider preferences in addressing it is a stepwise, stakeholder-engaged approach to support evidence-based cancer care. From this work, targeted improvement strategies to limit ADT overuse can be pursued.

### Supplementary Information

**Additional file 1:** STROBE Statement—Checklist of items that should be included in reports of cross-sectional studies

**Additional file 2:** Supplemental Table. COM-B domain statements across respondents

## Data Availability

The data sets supporting the conclusions of this article will be shared upon request. Members of the scientific community who would like a copy of the final data sets (i.e., data sets underlying any publication) from this study can request a copy by e-mailing Dr. Ted Skolarus at Ted.Skolarus@va.gov. The investigator should state their reason for requesting the data and their plans for analyzing the data. De-identified data will be provided after requestors sign a Letter of Agreement detailing the mechanisms by which the data will be kept secure and access restricted to their study team. The agreements will also state the recipient will not attempt to identify any individual whose data are included and will not share the data with anyone outside of their research team. The data set will not include private or protected information, and all dates will be changed to integers to allow for the calculation of time periods. Final data sets will be copied onto a CD and limited data sets will be encrypted, and the password will be sent to the requestor via e-mail. The CD will be sent to the requestor via FedEx. Each data set will be accompanied by documentation that lists all variables described in the publication and links them with variable names in the data set.

## Abbreviations

AACTT: Action, Actor, Context, Target, Time
ADT: Androgen deprivation therapy
COM-B: Capability, Opportunity, Motivation
DCE: Discrete choice experiment
PSA: Prostate-specific antigen
TDF: Theoretical domains framework
