# Supplementary material for: Unpacking overuse of androgen deprivation therapy for prostate cancer to inform de-implementation strategies
Source: Implement Sci Commun. 2024 Apr 9;5:37. doi: 10.1186/s43058-024-00576-x (PMC11005280; doi:10.1186/s43058-024-00576-x)
Supplement: Supplementary file 2 — Additional file 2: Supplemental Table. COM-B domain statements across respondents [file 43058_2024_576_MOESM2_ESM.docx]

**Supplemental Table. COM-B domain statements across respondents**

| **COM-B Domain and statement** | | **Strongly Agree/ Agree (%)** | |  |
| --- | --- | --- | --- | --- |
|  |  | Yes/  Probably Yes (n=61) | No/  Probably No  (n=23) | **p-value** |
| Opportunity – Social (patient), Motivation - Reflective | I find that patients are worried about the effect that stopping ADT will have on their cancer | 61% | 83% | 0.07 |
| Capability - Psychological | I find talking about stopping ADT challenging | 20% | 13% | 0.75 |
| Opportunity - Physical | I do not have adequate time for discussion about ADT | 16% | 4% | 0.28 |
| Opportunity – Social (provider) | I want to give ADT  recommendations consistent with  those of my peers | 75% | 91% | 0.14 |
| Opportunity - Environmental | I put a lot of weight on guideline  recommendations regarding use of  ADT as monotherapy (e.g., AUA or  NCCN) | 87% | 78% | 0.33 |
| Motivation - Reflective | I have concerns about side effects and castration resistance in patients with long-term use of ADT | 84% | 70% | 0.26 |
